# “*In situ similis*” Culturing of Plant Microbiota: A Novel Simulated Environmental Method Based on Plant Leaf Blades as Nutritional Pads

**DOI:** 10.3389/fmicb.2020.00454

**Published:** 2020-04-07

**Authors:** Rahma A. Nemr, Mohab Khalil, Mohamed S. Sarhan, Mohamed Abbas, Hend Elsawey, Hanan H. Youssef, Mervat A. Hamza, Ahmed T. Morsi, Mahmoud El-Tahan, Mohamed Fayez, Sascha Patz, Katja Witzel, Silke Ruppel, Kassem F. El-Sahhar, Nabil A. Hegazi

**Affiliations:** ^1^Environmental Studies and Research Unit, Department of Microbiology, Faculty of Agriculture, Cairo University, Giza, Egypt; ^2^Department of Microbiology, Faculty of Agriculture and Natural Resources, Aswan University, Aswan, Egypt; ^3^Regional Center for Food and Feed, Agricultural Research Center, Giza, Egypt; ^4^Algorithms in Bioinformatics, Center for Bioinformatics, University of Tübingen, Tübingen, Germany; ^5^Department of Plant Microbe Systems, Leibniz Institute of Vegetable and Ornamental Crops, Großbeeren, Germany; ^6^Department of Botany, Faculty of Agriculture, Cairo University, Giza, Egypt

**Keywords:** plant microbiota, plant-based culture media, endophyllosphere, endorhizosphere, MALDI-TOF-MS, culturomics, arid/semi-arid zones

## Abstract

High-throughput cultivation methods have recently been developed to accelerate the recovery of microorganisms reluctant to cultivation. They simulate *in situ* environmental conditions for the isolation of environmental microbiota through the exchange of growth substrates during cultivation. Here, we introduce leaf-based culture media adopting the concept of the plant being the master architect of the composition of its microbial community. Pre-physical treatments of sunflower plant leaves, namely punching, freezing, and/or autoclavation, allowed the diffusion of electrolytes and other nutrients to configure the leaf surface as a natural pad, i.e., creating an “*in situ similis*” environment suitable for the growth of rarely isolated microbiota. We used surface inoculation and membrane-filtration methods to assess the culturability of endophytic bacteria from the sunflower phyllosphere and rhizosphere. Both methods supported excellent colony-forming unit (CFU) development when compared to standard R2A medium, with a special affinity to support better growth of epiphytic and endophytic populations of the phyllosphere compared with the rhizosphere. A 16S rRNA gene analysis of >122 representative isolates indicated the cultivation of a diverse set of microorganisms by application of the new methods. It indicated the predominance of 13 genera of >30 potential species, belonging to Firmicutes, Proteobacteria, and Actinobacteria, and especially genera not commonly reported for sunflower, e.g., *Rhizobium*, *Aureimonas*, *Sphingomonas*, *Paracoccus*, *Stenotrophomonas*, *Pantoea*, *Kosakonia*, and *Erwinia*. The strategy successfully extended diversity and richness in the endophyllosphere compared to the endorhizosphere, while CFUs grown on the standard R2A medium mainly pertain to Firmicutes, especially *Bacillus* spp. MALDI-TOF MS analysis clustered the isolates according to their niche and potential functions, where the majority of isolates of the endorhizosphere were clustered away from those of the endophyllosphere. Isolates identified as Gammaproteobacteria and Alphaproteobacteria were distinguishably sub-clustered, which was in contrast to the heterogeneous isolates of Firmicutes (*Bacillus* spp.). In conclusion, leaf *in situ similis* cultivation is an effective strategy to support the future application of culturomics of plant microbiota. This is an effort to access novel isolates that are more adapted and competitive in their natural environments, especially those subjected to abiotic stresses like those prevailing in arid/semi-arid zones, and, consequently, to support the application of agro-biotechnologies, among other technologies, to improving agriculture in such zones.

## Introduction

Cultivation-dependent approaches are still indispensable for unlocking the treasure of environmental microbiomes for further elucidation of the ecology, physiology, and biotechnological applications of their major players (Aoi et al., 2009). Several attempts have been reported to improve the culturability of different environmental microbiota based on the modification of conventional cultivation methods and growth conditions. Among such attempts were the optimization of substrate compositions and concentrations (oligotrophic media), gelling agents, incubation time, cell density, trace additives of syntrophic growth factors, as well as signaling molecules. These efforts successfully improved the cultivation of rarely isolated bacterial phyla (Nichols et al., 2008, 2010). This is in addition to the high-throughput methods that have improved cultivation capabilities and recovered uncultivated microorganisms, including diffusion chambers (Kaeberlein et al., 2002; Bollmann et al., 2007), gel microdroplet-based microfluidic systems (Manome et al., 2001; Zengler et al., 2002), and microfluidic streak plates (Jiang et al., 2016), combined with a number of micro-devices (Ingham et al., 2005, 2007). These tools allowed the characterization of new ecologically important isolates (Morris et al., 2002; Zengler et al., 2002; Ferrari et al., 2005; Nichols et al., 2008).

The application of the concept of “environmental simulation” is most likely appropriate for the cultivation of environmental microbiomes, as multiple as yet unknown factors necessary for recovery and growth can be provided to the microorganisms (Ferrari et al., 2005). However, the development and use of the afore-mentioned methods and micro-devices are possibly limited due to the complexity of attaining the requisite technology and precision and to the relatively small scale of cultivation. They depend on the formation of μ-colonies, which require further a domestication/passage process to sustainably grow in standard Petri dishes (Nichols et al., 2010). Hence, there is a growing demand for the development of uncomplicated and easily applicable techniques that favor cultivation of microorganisms under *in vitro-*simulated environmental conditions mimicking to those *in situ*, i.e., “*in situ similis.*” Simulated environments enable the exchange of chemical compounds between the plant host and the colonizing microbiota during cultivation. The importance of creating *in situ* environmental conditions for the isolation of environmental microorganisms has been clearly demonstrated (Jung et al., 2016), and this need is very much amplified in the case of environments with adverse conditions, e.g., arid and semi-arid areas, where plant-associated microbiota modify their gene expression to cope with such stressed environments (Soussi et al., 2015). For this reason, we hereby introduce the use of intact leaf-based culture media for exploring plant microbiota. This method is based on the direct/or indirect inoculation and cultivation of microorganisms on the leaf surfaces in the form of culture pads, following the rationale that “natural environments envelop a multiplex of nutrients necessary for the growth of their inherent microbiota.” Hence, plant nutrients are supplied to the microorganisms in their natural/proportionate concentrations and gradients, provided that the leaves are pre-physically treated by punching, freezing, and/or autoclavation (Whitlow et al., 1992; Bajji et al., 2002; Verslues et al., 2006). This allows the recovery of microorganisms reluctant to cultivation because of inaccessibility and/or because the high concentrations of nutrients present in conventional cultivation media might be toxic to certain microorganisms. Most likely, the method favors slow-growing oligotrophic species that often follow the *k*-strategy growth model; these prefer nutrient-poor environments and are thought to be a major population in natural environments (Watve et al., 2000). As a natural habitat, the leaf pad was selected for our study to provide a suitable environment for co-culturing and to gain insight into interspecific/intraspecific interactions among the culturable community (Cleary et al., 2017). Throughout the present study, we investigated the cultivable community of bacteria residing in both the phyllosphere and rhizosphere compartments of sunflower plants. We compared the introduced leaf-based culture media to other forms of plant-based culture media recently presented by Sarhan et al. (2019) as well as the standard chemically synthetic R2A culture medium. To shed light on the diversity of the culturable community, representative isolates were subjected to 16S rRNA gene sequencing and matrix-assisted laser desorption/ionization time-of-flight mass spectrometry (MALDI-TOF MS) for identification and classification.

## Materials And Methods

### Plant Material and Sampling

The tested host plant was sunflower (*Helianthus annuus* L.), grown in the experimental fields of the Faculty of Agriculture, Cairo University, Giza, Egypt (30.0131°N, 31.2089°E) in a semi-arid environment^1^ (El-Ghani, 1998). Full-grown plants were sampled first by separation of the vegetative parts. The root system (intact roots with closely adhering soil) was then carefully uprooted. All samples were transferred to plastic bags. The microbiological analyses were carried out within 2 h of sampling.

The chemical compositions and nutritional contents of the tested sunflower plants were performed by the certified Regional Center for Food and Feed (RCFF), Agricultural Research Center (ARC), Giza, Egypt^2^. Analyses included total crude protein, total crude fiber, total ash, total carbohydrates, amino acids, as well as macro- and micro-nutrients.

Three preliminary experiments were carried out to test and adjust the most effective pre-physical treatments of plant leaves to allow outside leakage of nutrients to support the growth of inoculated bacteria on leaf surfaces. In addition, two main experiments were carried out to test the new leaf method for *in situ* recovery of bacteria: (1) bacteria extracted from the rhizosphere of sunflower plants and (2) bacteria extracted from the endorhizosphere and ecto- and endophyllosphere of sunflower plants. For each experiment, three plant samples, comprising plant roots and vegetative parts, were obtained to prepare composite samples for microbiological analysis.

### Culture Media

#### Leaf-Based Culture Media

Intact leaves were used in different preparations as the sole supportive substrate, i.e., culture pad, for culturing bacteria present in the various plant compartments of the rhizosphere and phyllosphere. Mature plant leaves were carefully washed by dipping in tap water several times, then once in distilled water. Leaves were blotted on filter paper for partial drying and cut into discs of 7–9 cm diameter to fit in Petri dishes. To encourage the leakage of nutrients and electrolytes out of such leaf discs, we applied the following protocols Whitlow et al., 1992 and Verslues et al., 2006). The leaf discs were either (i) punched by a metal vegetable grater, (ii) pressed several times by a meat hammer, and/or (iii) surface-scratched, making horizontal and vertical line cuts with a roller knife. These combinations of physical pre-treatments are tested as means to release nutrients and electrolytes from leaves to support *in vitro* bacterial growth. The prepared discs/pads were placed in Petri dishes (9 cm dia.) upside down to expose the lower surface of the leaf. Plates were subjected to overnight freezing at −20°C and then left to gradual thaw at ambient temperature (25–32°C) for several hours. The prepared plates were autoclaved at 121°C for 20 min; and upon cooling, soft water agar (1%; fine grade Oxoid agar, w/v) was poured into the plates to ensure a thick base layer of agar together with a thin overlay covering the leaf surface. Prepared agar plates were kept overnight at 25°C to allow diffusion of nutrients and electrolytes into the overlay agar as well as to check for sterility before use.

#### Plant-Teabag Culture Medium

The plant teabag culture medium was prepared according to the protocol described by Sarhan et al. (2016). Intact leaves of full-grown plants were initially washed in tap and distilled water. After being blotted on filter paper, they were dehydrated in the sun for 24 h, followed by oven drying at 70°C for 1–2 days. The dehydrated plant materials were mechanically ground to pass through a 2-mm sieve, obtaining fine dehydrated powder. Teabags were prepared by packing 0.5 g of the dehydrated powder into each bag and were closed by sealing with a stapler. One teabag of 0.5 g was soaked in 1 L of distilled water to obtain the liquid plant infusion. Agar culture media were prepared by adding agar (2%, w/v), pH-adjusted to 7.0, then autoclaved for 20 min at 121°C. The teabags were left in the culture media during autoclaving for further plant metabolite extraction. Media were tested to ensure sterility before use, as mentioned previously.

#### Standard Chemically Synthetic Culture Media

The standard culture media of nutrient agar (Jensen, 1962), R2A medium (Reasoner and Geldreich, 1985), and N-deficient combined carbon sources medium, CCM (Hegazi et al., 1998), were used. The standard nutrient agar consisted of 5 g l^–1^ peptone and 3 g l^–1^ beef extract, pH-adjusted to 7.2. The medium was used as such or diluted 1:10 (w/v). The R2A medium consisted of 0.5 g l^–1^ casein hydrolysate, 0.5 g l^–1^ dextrose, 0.5 g l^–1^ soluble starch, 0.5 g l^–1^ yeast extract, 0.3 g l^–1^ dipotassium hydrogen phosphate, 0.3 g l^–1^ sodium pyruvate, 0.25 g l^–1^ casein peptone, 0.25 g l^–1^ meat peptone, 0.024 g l^–1^ magnesium sulfate, and 20 g l^–1^ agar. The pH was adjusted to 7.2, and the culture medium was diluted to half strength and sterilized in an autoclave at 121°C for 20 min. The basal salt of CCM consisted of 0.4 g l^–1^ K_2_HPO_4_, 0.6 g l^–1^ KH_2_PO_4_, 0.2 g l^–1^ MgSO_4_, 0.1 g l^–1^ NaCl, 0.01 g l^–1^ MnSO_4_, 0.02 g l^–1^ CaCl_2_, 0.015 g l^–1^ FeCl_3_, 0.002 g l^–1^ Na_2_MoO_4_, 0.08 mg l^–1^ CuSO_4_, and 0.25 mg l^–1^ ZnSO_4_.

### Recovery of Bacteria Associated With Sunflower Roots and Leaves

In the case of the sunflower rhizosphere, ca. 5 g of roots, including closely adhering soil, were gently washed with tap water, then placed in a 45-ml diluent of basal salts of CCM and homogenized by shaking for 2 h at 150 rpm. This solution was referred to as the mother culture from which further serial dilutions were prepared. For the endorhizosphere samples, roots were surface-sterilized (Youssef et al., 2004) with 95% ethanol for 5–10 s. This was followed by 3% sodium hypochlorite for 30 min and then careful washing with sterilized distilled water before crushing in a Waring blender with an adequate volume of basal salts of CCM. Further serial dilutions were prepared for each of the whole rhizosphere and endorhizosphere samples. Dry weights were determined for suspended roots.

For the ectophyllosphere (epiphytic populations), ca. 5 g of intact sunflower leaves were put in flasks containing 50 ml of sterilized distilled water and shaken for 2 h at 150 rpm. For the endophyllosphere (endophytic populations), selected fresh leaves were washed with tap water, then surface-sterilized with ethanol (70% for 1 min) and sodium hypochlorite (3% for 5 min) and again with ethanol (70% for 1 min) (de Oliveira Costa et al., 2012; Jackson et al., 2013). Finally, the solutions were washed with sterilized water before crushing in a previously autoclaved Waring blender with adequate volumes of the basal salts of CCM as a diluent.

### Inoculation Methods

#### Leaf-Surface-Inoculation Method (LS)

Suitable serial dilutions of the tested plant rhizosphere (10^–2^:10^–5^) and phyllosphere (10^–1^:10^–4^) were selected for surface inoculation of prepared agar plates. Aliquots of 200 μl of each dilution were carefully spread on the overlay agar, covering the total leaf surface area. Plates were left right-side-up at ambient temperature for 1–2 h for the diffusion of inocula prior to incubation at 25–30°C. Incubation took place at 30°C for 2–10 days, and colony-forming units (CFUs) were counted.

#### Leaf-Membrane Filter Method (MF)

Aliquots of 1 ml of each of suitable dilutions (10^–1^:10^–4^) were mixed carefully with 5 ml distilled water prior to filtering through sterile 0.45-μm membrane filters (Sartorius AG, Germany). The prepared filters, containing the trapped bacterial cells, were carefully placed on the top of the overlay agar, covering the leaf surface area. Plates were left at ambient temperature for 1–2 h for the diffusion of inocula prior to incubation at 25–30°C. Incubation took place at 30°C for 2–10 days, and CFUs were counted.

For each method of leaf-based culture media treatment, the developed CFUs were examined visually and at 10-fold magnification using a stereomicroscope. In the case of the membrane filter method, membranes were quickly flushed by 1 ml of an aqueous methylene blue solution (1%, w/v) for better resolution.

### DNA Extraction and 16S rRNA Gene Analysis of Bacterial Isolates

To study the diversity of the culturable endophytes developed, 136 isolates were randomly selected by single colony isolation on corresponding culture media and represented the CFUs developed on all tested culture media (LS, MF, and R2A medium) (Supplementary Table S1). Bacterial genomic DNA was extracted from the isolates according to the method of Sarhan et al. (2016). DNA was extracted using the QIAGEN Dneasy Plant Mini Kit (Qiagen Inc., Hilden, Germany) according to the manufacturer’s instructions. The 16S rRNA genes were amplified with the forward primer “9bfm” (5′GAGTTTGATYHTGGCTCAG-3′) and reverse primer “1512R” (5′ACGGHTACCTTGTTACGACTT-3′) (Mühling et al., 2008). The PCR setup in a 25-μl volume was as follows: QIAGEN TopTaq Master Mix Kit 12.5 μl, PCR water 5.5 μl, primer 9 bfm (3.1 pmol μl^–1^) 2.5 μl, primer 1512R (3.1 pmol μl^–1^) 2.5 μl, and DNA (ca. 15 ng μl^–1^) 2.0 μl. The amplification of DNA was performed according to the thermal amplification cycling program: 4-min initial denaturing at 96°C, 30 thermal cycles of denaturation at 95°C for 1 min, annealing at 56°C for 1 min, and extension at 74°C for 1.5 min. The purified PCR products were sequenced by Eurofins MWG Operon, Ebersberg, Germany. Out of the 136 sequenced colonies, 122 were of sufficient lengths for further taxonomic and phylogenetic analyses.

### Protein Extraction From Bacterial Colonies and MALDI-TOF Mass Spectrometric Measurements

Protein-based characterization of isolates was performed *via* MALDI-TOF MS for 93 isolates of the leaf-based culture media, representing endophytes of roots (endorhizosphere) and shoots (endophyllosphere) as well as those of the ectophyllosphere. Briefly, samples of the tested isolates were grown on plant only-teabag culture medium at 28°C for 72 h. Then, a fine loop of bacterial cells was transferred into 300 μl of distilled water and 900 μl of absolute ethanol. The resulting suspension was pelleted by centrifugation at maximum speed (18,000 rpm) for 2 min at room temperature, dried for 2–3 min, and reconstituted in 1–80 μl of 70% formic acid, depending on the pellet size. One equal volume of each of acetonitrile and formic acid was added, mixing well. The resulting suspension was then centrifuged at maximum speed (18,000 rpm) for 2 min at room temperature. A volume of 1 μl of the supernatant was deposited directly onto a polished steel MALDI target plate (Bruker Daltonik GmbH, Bremen, Germany) and dried, and was then overlaid with 1 μl of matrix (Bruker Daltonik), which was a saturated solution of α-cyano-4-hydroxycinnamic acid in 50% acetonitrile/2.5% trifluoro-acetic acid. Intact protein masses were acquired using an ultrafleXtreme mass spectrometer (Bruker Daltonik) operating in linear positive mode. Acquisition of the mass spectra was performed in the range of 2.000–20.000 Da and at a sample rate of 1.2 GS s^–1^. The mass spectrometer was controlled by FlexControl version 3.4 software (Bruker Daltonik). The MALDI BioTyper software, version 3.1 (Bruker Daltonik), was used to process the raw spectra and to generate dendrograms.

### Statistical and Phylogenetic Analyses

The analysis of variance (ANOVA) and the least significant differences (LSD) were calculated using MSTAT-C software (Michigan State University, MI, United States). The R-package “ggplot2” was used for the construction of boxplots^3^.

For phylogenetic analyses, the 16S rRNA gene sequences were taxonomically assigned by comparison with those available in GenBank using BlastN (Altschup et al., 1990) and additionally with those of the Ribosomal Database Project (RDP) by using its classifier tool (Wang et al., 2007). Selected isolates and their closely related sequences (>10–20 sequences) were aligned with ClustalW2 (Thompson et al., 1994), and the phylogenetic trees were constructed by using the Neighbor Joining and Maximum Composite Likelihood methods, implemented in MEGA 7.0 (Kumar et al., 2016); bootstrapping was performed on 1000 replicates, and the inferred tree was saved in Newick format and visualized with iTol (Letunic and Bork, 2007). In the cases of potentially new isolates, phylogenetic analyses were further conducted in MEGA X, where similarity percentages were calculated according to the EZBiocloud database (Yoon et al., 2017).

To compare the 16S rRNA gene-based phylogenetic tree of the isolates with their corresponding protein profiles, derived from the MALDI BioTyper software, tanglegrams were generated using the R package “dendextend” version 1.9.0 (Galili, 2015). For the phyla Actinobacteria and Firmicutes or for Proteobacteria exclusively, we used the tanglegram function to convert each pair into its cladograms and to arrange the corresponding leaf labels in an optimal way, so that the crossing of the connecting lines between all the counterparts are minimized, while the splits remain. As the crossing of the connecting lines was considerably reduced when applying the tool Dendroscope 3 (Huson and Scornavacca, 2012), its layout was used as the template in the R script. In the final tanglegram, taxonomic affiliations are displayed at the leaf labels and colored according to the ranked class and genus. The inked bold leaf nodes specify the original isolation site (endorhizosphere or endophyllosphere) of the re-cultivated strains.

## Results

### Nutritional Profiles of the Tested Sunflower Leaves Used for the Preparation of Plant-Only-Based Culture Media

In order to characterize the nutritional matrix used in the preparation of plant-only-based culture media, the constitution of sunflower leaves was assessed, as shown in Table 1. Leaves were found to be rich in multiple nutrients required for the growth of the tested endophytic population, e.g., protein, carbohydrates, fat, fiber, ash, macro- and micronutrients, and amino acids.

**TABLE 1:** Chemical analyses of the dehydrated powder of leaves of the tested sunflower plants (*Helianthus annuus* L.).

**Parameters (%)**		**Amino acid (%)**	
Proteins	24.1	Aspartic (ASP)	2.13
Carbohydrates	50.31	Threonine (THR)	1.09
Fat	2.09	Serine (SER)	0.84
Crude fiber	7.96	Glutamic (GLU)	2.47
Ash	16.6	Proline (PRO)	0.92
Moisture	6.9	Glycine (GLY)	1.21
**Macro-nutrients (ppm)**		Alanine (ALA)	1.59
K	2.094	Valine (VAL)	1.61
Na	1.890	Methionine (MET)	0.57
Mg	0.224	Isoleucine (ILE)	0.94
Ca	2.49	Leucine (LEU)	1.88
P (%)	0.293	Tyrosine (TYR)	1.07
**Micro-nutrients (ppm)**		Phenylalanine (PHE)	1.29
Cu	2.52	Histidine (HIS)	0.36
Zn	0.389	Lysine (LYS)	0.96
Fe	3.12	Arginine (ARG)	1.16
Mn	1.342	Cysteine (CYS)	0.29
Se (ppb)	68.72		
Pb (ppb)	0.234		

### Physical Pre-treatments of Plant Leaves to Release Nutrients and Electrolytes for *in situ similis* Cultivation of Sunflower-Associated Microbiota

**FIGURE 1:**
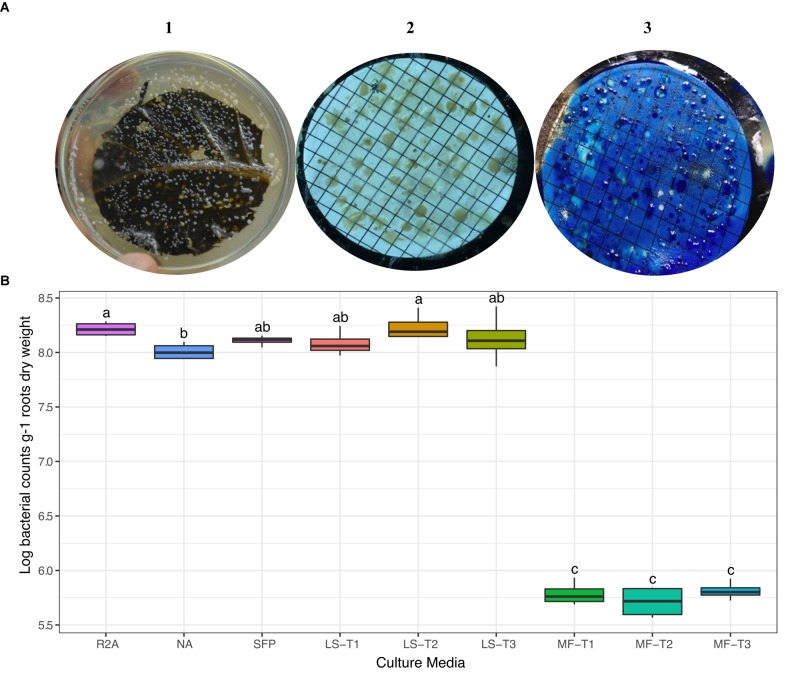
**(A)** Colony morphology of bacteria isolated from the rhizosphere of sunflower plants cultured on different culture media. CFUs developed on the leaf surface-inoculated culture medium (1) and by the leaf-membrane filter method (2); notice the better resolution of colonies when membrane filters were stained with methylene blue (3). **(B)** ANOVA test of log CFU counts of rhizobacteria recovered by standard culture media (R2A medium; nutrient agar, NA; sunflower teabag, SFP) and leaf-based culture media (LS, leaf-surface-inoculated; MF, leaf-membrane filter). Physical leaf pre-treatments: punching, T1; punching and pressing, T2; punching, pressing, and scratching, T3. Statistically significant differences (LSD) are indicated by different letters (*P* value 0.05).

We carried out preliminary experiments to evaluate the efficiency of different physical leaf pre-treatments and inoculation methods (LS and MF). Physical pre-treatments of leaves, by punching, pressing, and/or scratching, were exercised to allow outside release of nutrients and electrolytes to support the growth of superposed bacterial inocula. In general, there was no statistical difference in CFU counts between the different leaf pre-treatment conditions (Figure 1); however, the combined effect of punching, pressing, and scratching resulted in a higher diversity of colony morphologies. CFUs developed nicely on the leaf-based culture media, either surface-inoculated leaves or prepared membrane filters (Figure 1A). Colonies differed in shape (circular, irregular), size (1–5 mm dia.), consistency (watery, paste-like, and leathery), elevation (flat, raised, and convex), opacity (opaque), and color (white, creamy, and yellow). Direct surface inoculation on the top of leaf surfaces was superior to the indirect placement of prepared membrane filters on the leaf surfaces. This method was supportive for CFU development (>log 8.0 g^–1^), very much comparable to the tested standard culture media (diluted nutrient agar and R2A) as well as the other plant-based culture media in the form of teabags (Figure 1B).

Possibilities of contamination were checked during these preliminary experiments over a longer incubation period. It appeared that contamination, if any, appeared due to external sources as early as during the first day of incubation, in the form of big (>3 mm), flat, spread white to creamy colonies. When microscopically examined, they showed a form different from bacilli cells. Therefore, as a routine check for contamination throughout the following experiments, we discarded prepared leaf-agar plates that showed any contamination during the first day of incubation.

### Bacterial CFU Counts of Rhizosphere (Endorhizosphere) and Phyllosphere (Ecto- and Endophyllosphere) as Developed on Leaf-Based and Standard Culture Media

**FIGURE 2:**
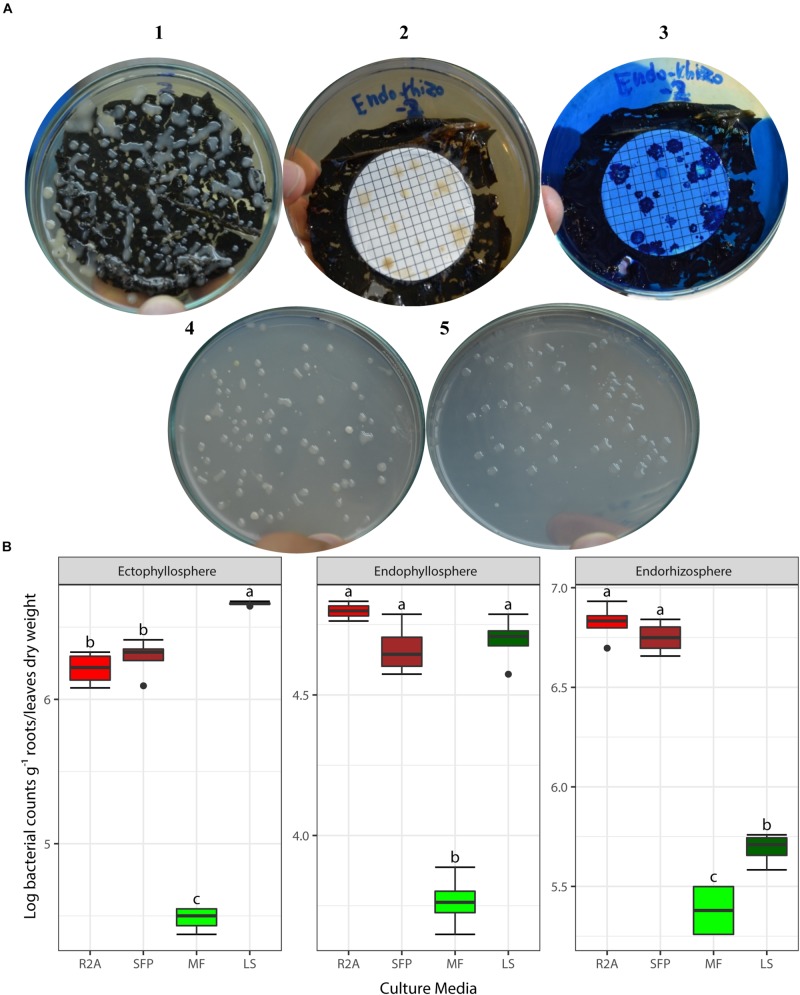
**(A)** CFUs developed from culturing bacteria present in the endorhizosphere of sunflower plants. Colonies grown with both the leaf surface-inoculation (1) and leaf-membrane filter methods with and without staining (2, 3) compared to colonies developed on standard R2A medium (4) and plant-only-teabag culture media (5). **(B)** Two-way ANOVA, interaction of plant sphere and culture media: Log CFU counts recovered by the standard culture media (R2A medium and sunflower teabag, SFP) compared to the leaf-based culture media (MF, leaf-membrane filter; LS, leaf-surface-inoculated). Statistically significant differences (LSD) are indicated by different letters (*P* value 0.05).

Based on the results obtained from the preliminary experiments, we carried out two main experiments, employing the combined physical pre-treatments of leaf-based culture media to support the recovery of the *in situ* bacterial populations found in the endorhizosphere and ecto- and endophyllosphere of the sunflower. The leaf-based method was compared to both R2A medium and the plant powder teabag culture medium. The results indicated statistically significant differences attributed to the single effects of incubation time, type of culture media, and plant sphere (Table 2). In general, and irrespective of plant sphere, surface-inoculated leaf-based culture medium supported growth and development of CFUs that was very much comparable to that supported by both R2A and plant-teabags culture media (Figure 2). Longer incubation up to 10 days resulted in significant increases in CFU numbers for surface-inoculated leaves but not for the membrane filter method (Table 2). Of interest is that the leaf surface-inoculation method, when compared to all tested culture media, showed affinity to support better growth of synonymous epiphytic and endophytic populations of the phyllosphere. Additionally, the method supported good culturability (log 5.7 CFUs g^–1^) of bacteria in the endorhizosphere; however, higher counts were reported for the plant-only-teabag culture medium and R2A medium (>log 6.0 CFUs g^–1^).

**TABLE 2:** One-way ANOVA test of CFU counts of the endorhizosphere, ectophyllosphere, and endophyllosphere from sunflower plants: single effects of incubation time, type of culture media, and plant sphere.

**Treatment**	**log CFUs g^–1^ root or leaf**
**(Incubation time)**	
2 days	5.02^c^
4 days	5.36^b^
10 days	5.52^a^
**LSD (*P*-value ≤ 0.05) = 0.046**	
**(Culture media)**	
R2A (half strength)	5.80^a^
Sunflower teabags (0.5g l-1)	5.66^b^
Punched + pressed + scratched - leaf-surface-inoculated	5.45^c^
Punched + pressed + scratched - leaf-membrane filter	4.29^d^
**LSD (*P*-value ≤ 0.05) = 0.053**	
**(Plant spheres)**	
Endorhizosphere	6.03^a^
Endophyllosphere	4.23^c^
Ectophyllosphere	5.64^b^
LSD (*P*-value ≤ 0.05) = 0.046	

*Statistically significant differences (LSD) are indicated by different letters (*P*-value ≤ 0.05).*

### Bacterial Community Composition Based on 16S rRNA Gene Sequencing of Representative Isolates

**FIGURE 3:**
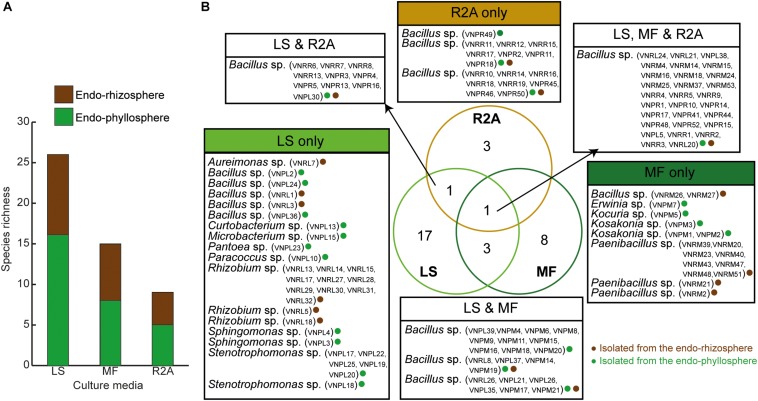
**(A)** Richness of endophytic populations present in the endorhizosphere and endophyllosphere of sunflower plants, grown on the tested culture media; **(B)** Venn diagram representing potential species among the tested culture media as affected by the extracted plant spheres. LS, leaf surface-inoculated; MF, leaf-membrane filter; R2A, Reasoner’s 2A culture medium. The strain ID of isolates belonging to the related potential species appear in brackets.

**FIGURE 4:**
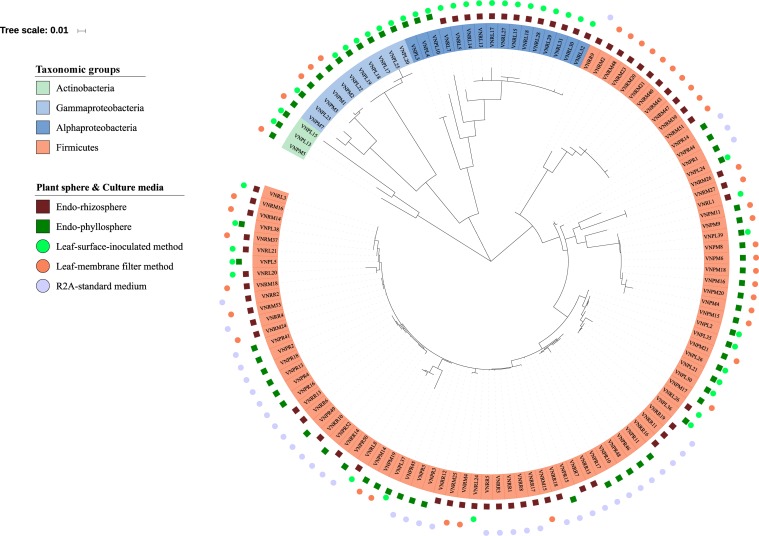
Phylogenetic tree of 16S rRNA gene sequences of the 122 isolates obtained. The colored labels indicate the taxonomic groups (Actinobacteria, Gammaproteobacteria, Alphaproteobacteria, Firmicutes), plant compartments (endorhizosphere 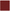 and endophyllosphere 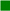), and culture media (leaf-surface-inoculation method 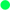, leaf-membrane filter method 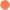, and standard R2A culture medium 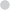).

Out of a total of 333 CFUs randomly selected on all tested culture media, 122 were progressively subcultured and produced good quality sequences. They developed on LS, MF, and R2A culture media for the plant compartments endorhizosphere (20, 21, and 19 isolates, respectively) and endophyllosphere (23, 18, and 21 isolates, respectively, Supplementary Table S1). Based on the 16S rRNA gene sequence alignment, those isolates were classified into 13 genera, of a potential 30 species, and three bacterial phyla (Supplementary Tables S2 and S3). Some of these taxa were represented by a single cultivated isolate, while others contained multiple ones. Higher numbers of genera and of potential species were found to be exclusively cultivated by either leaf surface-inoculation or leaf-membrane filter method, while only three potential species of *Bacillus* sp. were confined to growth on R2A-standard culture medium (Figure 3). The genus *Bacillus* sp., with five potential species, were particularly overlapped among all of the tested culture media (Figure 3B). The culturable sunflower-associated bacteria were represented by the three phyla of Firmicutes, Proteobacteria, and Actinobacteria, with corresponding relative abundance in descending order of 75, 22, and 3%, respectively. We compared the richness (Figure 3A) and relative abundance (Figure 3B) of various phyla and genera in both the endorhizosphere and the endophyllosphere. In general, the standard R2A culture medium showed an overwhelming abundance of Firmicutes, represented by *Bacillus* spp., in both compartments. The picture completely differed with the two leaf-based culture media, where in the endorhizosphere, the relative abundance of Firmicutes was 50–100% (*Bacillus* spp. and *Paenibacillus* spp.) followed by Alphaproteobacteria (0–50%; *Aureimonas* spp. and *Rhizobium* spp.). Higher diversity was reported in the endophyllosphere, where there were four taxonomic groups, Firmicutes (44–50%; *Bacillus* spp.), Gammaproteobacteria (29–44%; *Stenotrophomonas* spp., *Pantoea* spp., *Kosakonia* spp., and *Erwinia* spp.), Alphaproteobacteria (0–13%; *Sphingomonas* spp. and *Paracoccus* spp.), and Actinobacteria (8–11%; *Curtobacterium* spp., *Microbacterium* spp., and *Kocuria* spp.), in descending order. The overall clustering of total isolates according to their taxonomic position, plant compartments, and culture media is illustrated in Figure 4.

### Clustering of Bacterial Isolates Based on Whole-Cell MALDI-TOF MS Analysis

We set out to use MS-based bio-typing to classify successfully sequenced bacterial isolates, particularly those that developed on leaf-surface-inoculated and leaf-membrane filter culture medium and originated from the endorhizosphere and endophyllosphere (Supplementary Table S2). The intact protein mass spectra of 62 isolates were assessed for similarity. The results obtained showed that the analyzed isolates clustered into two main groups (Supplementary Figure S1). The first main group contained most of the isolates, 17 out of 23, representing 73.9%, originated from the endorhizosphere. Relying on the identification based on 16S rRNA gene sequencing, this first main group was subdivided into three clusters, two of which were mainly occupied by representatives of Firmicutes (*Bacillus* spp.) and the third of which was confined to Alphaproteobacteria (*Rhizobium* spp.), except for a single isolate of *Bacillus subtilis*. It is of interest that the cluster of *Rhizobium* spp. principally developed only on the leaf-surface-inoculated culture medium, while the other two clusters of fast-growing *Bacillus* spp. occupied leaf-based membrane filter culture medium. On the other hand, the second main group contained the majority of endophyllosphere isolates, 28 out of 39, representing 71.8%. Within this main group, Gammaproteobacteria (*Stenotrophomonas* spp. and *Kosakonia* spp.) and Alphaproteobacteria (*Aureimonas* spp. and *Paracoccus* spp.) were sub-clustered, contrary to the many heterogeneous members of Firmicutes (*Bacillus* spp.), which were scattered among the subsequent seven sub-clusters. Again, representatives of Firmicutes were mostly isolated on leaf-based membrane filter, while all the genera representing Actinobacteria, Alphaproteobacteria, and Gammaproteobacteria exclusively developed on the leaf-surface-inoculated culture medium. It appeared that representatives of Firmicutes, being fast-growers, did occupy and overcome the limited available surface area of the membrane filters, not giving representatives of other phyla the opportunity to develop with prolonged incubation.

**FIGURE 5:**
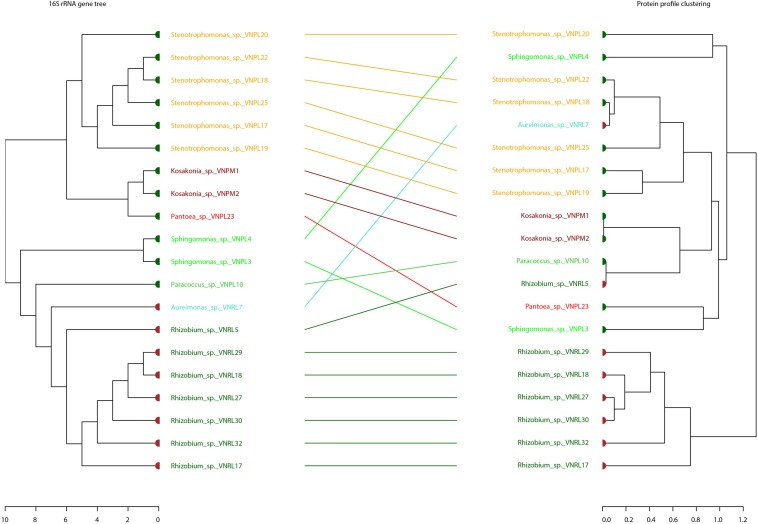
Comparison of the phylogenetic 16S rRNA gene and MALDI bio-typer protein profile clusters: tanglegrams for the phyla Proteobacteria were generated with the R package dendextend and the tool Dendroscope 3 to display the phylogenetic (left) and protein pattern-specific clusters (right) in an aligned style. Colored leaf labels indicate the group species name and taxonomic common affiliation, while the colors of the bold leaf nodes specify the isolation site (green: endophyllosphere, brown: endorhizosphere) to emphasize (dis-) similarities. Indicated names of species are potential/putative, based on partial 16S rRNA gene sequencing, and one should exercise caution in relying on names of genera.

Binary tanglegrams were constructed to demonstrate one-to-one correspondence between the phylogenetic classification and protein profiles of the tested isolates. While all Proteobacteria isolates (Figure 5, 20 isolates) clustered according to their phylogenetic relationships based on 16S rRNA gene amplicons, i.e., Alphaproteobacteria and Gammaproteobacteria and their correspondent genera, no clear separation was reported for their protein profiles due to the overlapping protein expression patterns of five Alphaproteobacteria isolates (VNPL4; VNRL7; VNPL10; VNPL3; and VNRL5). However, both classes evince distinguishable protein profile clusters. Regarding the impact of the culture media and plant sphere on the protein pattern, we can demonstrate a separation due to the plant spheres endorhizosphere and endophyllosphere, except for the two strains of *Aureimonas altamirensis* VNRL7 and *Rhizobium rosettiformans* VNRL5. In contrast, the evolutionary grouping/clustering of Actinobacteria and Firmicutes (42 isolates) was not reflected in the protein profile. Likewise, on the genus level, distinct clusters of protein profiles were absent, although some minor phylogenetic sub-clades reappeared. While strains did not group specifically according to their culture media, plant sphere-specific sub-clusters were observed.

## Discussion

Our previous investigations, recently reviewed by Sarhan et al. (2019), provided evidence that plant materials, such as crude slurry homogenates, juices, saps, and dehydrated powders, as such, without any supplement, can be successfully used to enhance the culturability of plant-associated microbiota. In the present study, we further advance the leaf-based culture strategy for the growth of epiphytic and endophytic microbiota of sunflower grown in fields in Giza, Egypt, in semi-arid land environments. The principle applied allows the exchange of and access to multiple plant nutrients in their natural/proportionate concentrations and gradients. Direct (LS) or indirect contact (MF) of the inocula to the leaf surface creates an *in situ similis* environment where bacterial cells get access to their natural and complex nutritional matrices of macro-molecules, macro- and micro-nutrients, and amino acids (Table 1). We consider that this set up enables the recovery of slow-growing microorganisms reluctant to cultivation, which often follow *k*-strategy, being either of prototrophic or auxotrophic nature, and are thought to be a major intrinsic component in natural environments.

The culturing strategy introduced supported good recovery of bacteria associated with the various plant compartments, very much challenging the tested R2A standard culture media. The novel plant-based media provided excellent growth of CFUs of epiphytic and endophytic populations of the phyllosphere, as well as for those present in the endorhizosphere. Most of the isolated strains were unique to their isolation approach. This indicates that the “*in situ similis* leaf cultivation” strategy introduced substantially increased the overall diversity and richness in the endophyllosphere compared to the endorhizosphere. Here, one might expect the potential interaction of both biotic factors, e.g., host species, genotype, and leaf age, as well as abiotic factors, e.g., drought and salt stress in arid regions. They all influence the structure of microbial communities associated with plants (Redford and Fierer, 2009). The stress imposed does vary depending upon the location of the microbial community within the plant, and this may have repercussions in terms of the structure and function of microbial communities. For example, microbes residing in the phyllosphere are faced with a nutrient-poor and variable environment that is characterized by fluctuating temperature, humidity, and UV radiation (Lindow and Brandl, 2003). The microbial community in the rhizosphere, on the other hand, resides within a relatively stable environment that is rich in nutrients due to the chemicals exuded by plants to recruit beneficial microorganisms and counteract pathogens (Badri et al., 2009). However, there are also cases where bacteria from the soil first colonize the roots and then migrate to the above-ground part of the plant, indicating that those microorganisms are well adapted to multiple plant-affected environments (Czajkowski et al., 2009; Bodenhausen et al., 2013; Leach et al., 2017; Hassani et al., 2018).

While the standard R2A medium fostered the growth of the single genus of *Bacillus* spp., the leaf cultivation strategy was unique in extending the diversity of culturable plant microbiota. The strategy recovered 13 genera representing the three major phyla of Firmicutes, Proteobacteria, and Actinobacteria. Representatives of these phyla are commonly reported in different compartments of plants subjected to various environments, including both biotic and abiotic stresses (Koberl et al., 2011; Marasco et al., 2012; Sarhan et al., 2016, 2018; Hegazi et al., 2017).

Based on a literature survey on sunflower-associated bacteria, we compared the outcome of our leaf cultivation strategy with those of other culture-dependent techniques (Table 3). Similarly, the leaf cultivation strategy successfully recovered genera representing the big phyla of Firmicutes and Proteobacteria. In contrast to this, our strategy of extended cultivation led to the isolation of three genera of Actinobacteria (*Curtobacterium*, *Microbacterium*, and *Kocuria*). This is in conformity with our recent report on the significant enrichment of such genera of Actinobacteria on plain water agar media following an inoculum-dependent culturing, where the administered plant inoculum is the only source of nutrients for the growth of plant endophytes (Sarhan et al., 2020). As to the genera representing the phyla of Alphaproteobacteria and Gammaproteobacteria, the leaf cultivation strategy further enriched genera not reported earlier in the compartments of sunflower plants, e.g., *Rhizobium* sp., *Aureimonas* sp., *Sphingomonas* sp., *Paracoccus* sp., *Stenotrophomonas* sp., *Pantoea* sp., *Kosakonia* sp., and *Erwinia* sp. (Table 3). Such a rich diversity of the sunflower microbiome was further confirmed by culture-independent techniques (e.g., Alsanius et al., 2017 and Oberholster et al., 2018), with particular enrichment of the key genera *Rhizobium* sp., *Sphingomonas* sp., *Burkholderia* sp., and *Pseudomonas* sp. In the present study, we isolated two of those key genera, i.e., *Rhizobium* sp. and *Sphingomonas* sp., which are considered as hub taxa and play a central role in the assembly of the sunflower microbiome throughout its life cycle (Leff et al., 2016; Alsanius et al., 2017; Oberholster et al., 2018). The other two genera of *Burkholderia* sp. and *Pseudomonas* sp. were particularly enriched by a modified leaf cultivation method using MPN tubes of semi-solid water agar with immersed leaf strips into which research is ongoing (unpublished data).

**TABLE 3:** Literature related to the diversity and community composition of sunflower microbiota as recovered by various culture-dependent techniques and culture media.

**References**	**Cultivation methods**	**Detected phyla/families/genera/species**
Ambrosini et al., 2016	**Culture-dependent:** • Isolation from rhizospheric soils. • CFU counts on N-free thiamine–biotin agar medium (TB).	• **Total of 101 strains were identified.** • Based on 16S rRNA gene sequences, isolates belonged to the genera *Bacillus* spp. and *Paenibacillus* spp., represented by 41.6 and 58.4%, respectively. • **Isolates of *Bacillus* (42 isolates):** *B. arbutinivorans, B. cereus, B. aryabhattai, B. drentensis, B. pumilus, B. safensis, B. acidiceler, B. nealsonii*, and *B. mycoides*. • ***Paenibacillus* (59 isolates):** *P. pabuli, P. taichungensis, P. tundrae, P. amylolyticus, P. xylanexedens, P. barcinonensis, P. illinoisensis, P. xylanilyticus, P. durus, P. typhae, P. catalpa, P. glycanilyticus, P. jilunlii, P. sonchi, P. graminis*, and *P. riograndensis.*
Campos et al., 2012	**Culture-dependent:** • Isolation from different plant tissues (roots, stems, florets, rhizosphere). • Semi-solid N-free media (LGI, NFb, JMV, and Dygs). • MPN and CFU counts.	• **Total of 57 PGP isolates, 45 of which were identified.** • Based on 16S rRNA gene sequencing, 42 belonged to *Bacillus* spp. (*B. subtilis*, *B. cereus*, *B. thuringiensis*, *B. pumilus*, *B. megaterium*, *Bacillus* sp.) and three to *Methylobacterium komagatae*.
Forchetti et al., 2007	**Culture-dependent:** • Endophytic bacteria isolation from roots of sunflowers grown under stress conditions.	• **Total of 29 endophytic strains were isolated.** • Among them, eight strains have PGP functions: one showed 99.9% sequence homology with *Achromobacter xylosoxidans* or *Alcaligenes* sp., and seven showed 99.9% homology with *Bacillus pumilus*.
Liu et al., 2017	**Culture-dependent:** • Isolated from the rhizosphere soil. • Nitrogen-free Ashby medium.	• **Total of 11 strains were isolated.** • One *Enterobacter cowanii*, two *E. ludwigii*, two *E. cancerogenus*, three *Klebsiella oxytoca*, *Pseudomonas lini*, and one *Bacillus megaterium*/*aryabhattai.*
Raval and Desai, 2012	**Culture-dependent:** • Isolated from various regions of the sunflower plant (bulk soil, rhizosphere, and endorhizosphere). • Specialized media for PGP bacteria, namely *Azotobacter*, *Pseudomonas*, and *Bacillus* species.	• **Total of 30 isolates were obtained.** • Isolates belonged to *Azotobacter*, *Pseudomonas*, and *Bacillus* species. • Seven possessed several plant growth-promoting activities.

**FIGURE 6:**
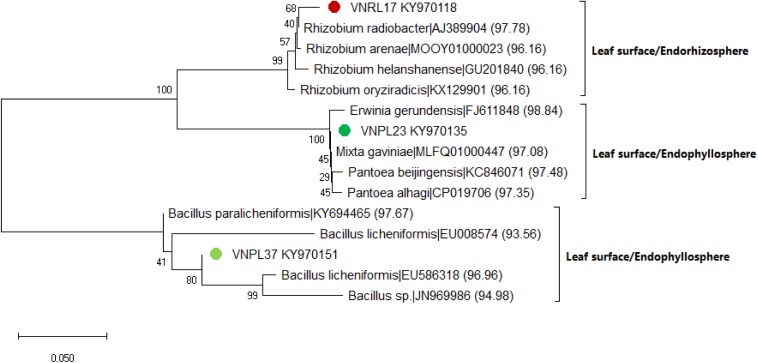
Maximum likelihood (ML) phylogenetic tree of the bacterial isolates recovered by the leaf surface-inoculation method from the endorhizosphere (VNRL17) and endophyllosphere (VNPL23, VNPL37) of sunflower plants, based on 16S rRNA gene sequences. Bootstrapping was performed for each tree with 1000 replicates; the percentage of trees in which the associated taxa clustered together is shown next to the branches; numbers between brackets refer to the similarity percentage according to the EZBiocloud database.

The leaf cultivation strategy recovered three isolates (VNRL17, VNPL23, and VNPL37) that might represent putative novel species, as their highest matching scores were <98.7%. This was further confirmed by maximum likelihood (ML) phylogeny, which displayed their significant separation from all the deposited members of the related genera (*Rhizobium* sp., *Erwinia* sp., and *Bacillus* sp., Figure 6). The relative abundance of reported genera/species was different among the two methods of leaf-based culture; Firmicutes dominated the pool of the indirect membrane filter-derived strains (MF, 86.8%), and Proteobacteria dominated the pool of direct leaf-surface strains (LS, 53.5%). Further molecular and physiological analyses are being carried out to confirm their novelty.

Matrix-assisted laser desorption/ionization time-of-flight mass spectrometry-based microbial characterization has become a valuable tool for the fast and accurate classification of isolates using the intact protein pattern as a fingerprint. The method is widely applied, especially for the human microbiome, for re-replication of recurrent isolated strains and for their identification (Huschek and Witzel, 2019). Our results indicate that MALDI-TOF MS is a suitable tool for the reliable characterization of protein profiles for certain, not all, groups of microorganisms, as in the case of identification/clustering of *Rhizobium* spp., family Rhizobiaceae. This is in accordance with earlier reports by a number of investigators (Ferreira et al., 2011; Sánchez-Juanes et al., 2013). All six isolates of *Rhizobium* spp. are clustered together and have comparable protein mass spectra. This contrasts with the many different spectra of *Bacillus* spp., which were heterogeneous enough to be scattered among many sub-clusters. It is already reported that *Bacillus* spp. contains more than 80 valid species that are very closely related, difficult to discriminate, and have a high percentage of similarity (over 99%) on the basis of 16S rRNA gene sequencing identification. However, they are different according to their functional properties, including pathogenicity (Starostin et al., 2015). In general, the leaf-based culture strategy has the potential to cluster isolates according to their niche and potential functions. The isolates of the endorhizosphere were clustered in one of the two main groups away from the second group containing the isolates of the endophyllosphere origin. The tanglegrams constructed emphasize the potential of MALDI-TOF MS-based analyses of protein profile clustering in combination with cross-cultivation on the leaf nutritional pad for identification of isolates that reveal specific functions, e.g., colonizing specific plant niches. This conclusion is supported by the finding that even phylogenetically distant species that colonize the same or different plant spheres reveal similar protein profiles on the same nutritional media (Katja Witzel, personal communication). Although an overall separation of protein profile clusters is partly given for the classes Alpha- and Gammaproteobacteria, taxonomic or phylogenetic comparability must be regarded with caution and needs to be examined in detail, and much more so when comparing across phyla. One reason may be that the limited phylogenetic analysis used in our approach is based only on one phylogenetic marker gene, whereas the protein-derived clusters comprise the occurrence and quantity patterns of multiple proteins under a given cultivation method/environment (Sauget et al., 2017).

## Conclusion

In conclusion, there is a wide range of evidence to show that the natural resources of arid/semi-arid regions are suffering serious damage as they face increasing demands and mounting pressures^5^. Here, the need arises to search for and adopt agro-biotechnologies, among other technologies, to improving agriculture in such regions. To yield hidden and compatible new resources of microbiota pertinent to the agro-biotechnologies approved, we recommend the strategy of “leaf *in situ similis* cultivation” introduced here. Moreover, this strategy, in particular, and plant-only-based culture medium, in general, are powerful approaches to extend the application of culturomics techniques. This is in an effort to understand the ecology and physiology of uncultivable members of plant microbiota subject to varied abiotic stresses, e.g., those of heat and drought occurring in arid/semi-arid climatic zones (Soussi et al., 2015).

## Data Availability Statement

The datasets generated for this study (partial 16S rRNA gene sequences, 559–1030 bp) have been deposited in the GenBank database under the accession numbers KY970089–KY970106, KY970108–KY970127, KY970129–KY970151, KY953159–KY953179, MG920148–MG920166, and MG952230–MG952264.

## Author Contributions

NH conceived of the idea, developed the theory, and followed the experimental program. SR and KE-S were involved in planning and supervising the experimental work. RN carried out the experiments and results analysis. KW supervised MALDI-TOF experiments. SP carried out the bioinformatics analysis. ME-T supervised the chemical analysis of tested plant materials. HY, MA, and MH performed the statistical analysis. MS supported the lab work and interpretation of the results. AM and MK contributed to the sample preparation and experimentation. HE provided the technical support. RN, NH, SR, and MF wrote the manuscript, with contributions from all authors.

## Conflict of Interest

The authors declare that the research was conducted in the absence of any commercial or financial relationships that could be construed as a potential conflict of interest.

## Footnotes

1 https://unesdoc.unesco.org/ark:/48223/pf0000032661

2 rcff.com.eg/ISO%20Accriditation/Scope.htm

3 ggplot2.tidyverse.org

5 http://www.fao.org/3/t0321e/t0321e-08.htm

## References

[B1] Alsanius B. W. Bergstrand K. Hartmann R. Gharaie S. Wohanka W. Dorais M. (2017). Ornamental flowers in new light: artificial lighting shapes the microbial phyllosphere community structure of greenhouse grown sunflowers (*Helianthus annuus* L.). *Sci. Hortic.* 216 234–236. 10.1016/j.scienta.2017.01.022

[B2] Altschup S. F. Gish W. Pennsylvania T. Park U. (1990). Basic Local Alignment Search Tool. *J. Mol. Biol.* 215 403–410. 10.1016/S0022-2836(05)80360-2 2231712

[B3] Ambrosini A. Stefanski T. Lisboa B. B. Beneduzi A. Vargas L. K. Passaglia L. M. P. (2016). Diazotrophic bacilli isolated from the sunflower rhizosphere and the potential of *Bacillus mycoides* B38V as biofertiliser. *Ann. Appl. Biol.* 168 93–110. 10.1111/aab.12245

[B4] Aoi Y. Kinoshita T. Hata T. Ohta H. Obokata H. Tsuneda S. (2009). Hollow-fiber membrane chamber as a device for in situ environmental cultivation. *Appl. Environ. Microbiol.* 75 3826–3833. 10.1128/AEM.02542-08 19329655 PMC2687273

[B5] Badri D. V. Weir T. L. Lelie D. Van Der Vivanco J. M. (2009). Rhizosphere chemical dialogues: plant – microbe interactions. *Curr. Opin. Biotechnol.* 20 642–650. 10.1016/j.copbio.2009.09.014 19875278

[B6] Bajji M. Kinet J. Lutts S. (2002). The use of the electrolyte leakage method for assessing cell membrane stability as a water stress tolerance test in durum wheat. *Plant Growth Regul.* 36 61–70. 10.1023/A:1014732714549

[B7] Bodenhausen N. Horton M. W. Bergelson J. (2013). Bacterial communities associated with the leaves and the roots of *Arabidopsis thaliana*. *PLoS One* 8:e0056329 10.1371/journal.pone.0056329 PMC3574144 23457551

[B8] Bollmann A. Lewis K. Epstein S. S. (2007). Incubation of environmental samples in a diffusion chamber increases the diversity of recovered isolates. *Appl. Environ. Microbiol.* 73 6386–6390. 10.1128/AEM.01309-07 17720826 PMC2075052

[B9] Campos K. Pinheiro G. Luisa M. Fisher D. C. Cattelan A. J. Nogueira M. A. (2012). Biochemical and molecular characterization of high population density bacteria isolated from Sunflower. *J Microbiol Biotechnol.* 22 437–447. 10.4014/jmb.1109.09007 22534289

[B10] Cleary J. L. Condren A. R. Zink K. E. Sanchez L. M. (2017). Calling all hosts: bacterial communication in situ. *Chem.* 2 334–358. 10.1016/j.chempr.2017.02.001 28948238 PMC5609483

[B11] Czajkowski R. de Boer W. J. Velvis H. Van Der Wolf J. M. (2009). Systemic colonization of potato plants by a soilborne, green fluorescent protein-tagged strain of *Dickeya* spp. *Biovar* 3. *Phytopathology* 100 134–142. 10.1094/PHYTO-100-2-0134 20055647

[B12] de Oliveira Costa L. E. de Queiroz M. V. Borges A. C. de Moraes C. A. de Araújo E. F. (2012). Isolation and characterization of endophytic bacteria isolated from the leaves of the common bean (*Phaseolus vulgaris*). *Braz. J. Microbiol.* 43 1562–1575. 10.1590/S1517-838220120004000041 24031988 PMC3769033

[B13] El-Ghani M. M. A. (1998). Environmental correlates of species distribution in arid desert ecosystems of eastern Egypt. *J. Arid Environ.* 38 297–313. 10.1006/jare.1997.0323

[B14] Ferrari B. C. Binnerup S. J. Gillings M. (2005). Microcolony cultivation on a soil substrate membrane system selects for previously uncultured soil bacteria. *Appl. Environ. Microbiol.* 71 8714–8720. 10.1128/AEM.71.12.8714-8720.2005 16332866 PMC1317317

[B15] Ferreira L. Sánchez-Juanes F. García-Fraile P. Rivas R. Mateos P. F. Martínez-Molina E. (2011). MALDI-TOF mass sppectrometry is a fast and reliable platform for identification and ecological studies of sppecies from family rhizobiaceae. *PLoS One* 6:e20223 10.1371/journal.pone.0020223 PMC3105015 21655291

[B16] Forchetti G. Masciarelli O. Alemano S. Alvarez D. (2007). Endophytic bacteria in sunflower (*Helianthus annuus* L.): isolation, characterization, and production of jasmonates and abscisic acid in culture medium. *Appl. Microbiol. Biotechnol.* 76 1145–1152. 10.1007/s00253-007-1077-7 17657487

[B17] Galili T. (2015). dendextend: an R package for visualizing, adjusting, and comparing trees of hierarchical clustering. *Bioinformatics* 31 3718–3720. 10.1093/bioinformatics/btv428 26209431 PMC4817050

[B18] Hassani M. A. Durán P. Hacquard S. (2018). Microbial interactions within the plant holobiont. *Microbiome* 6 1–17. 10.1186/s40168-018-0445-0 29587885 PMC5870681

[B19] Hegazi N. A. Hamza M. A. Osman A. Ali S. Sedik M. Z. (1998). “Modified combined carbon N-deficient medium for isolation, enumeration and biomass production of diazotrophs,” in *Nitrogen Fixation With Non-Legumes*, eds Malik K. A. Mirza M. S. Ladha J. K. (Dordrecht: Springer), 247–253. 10.1007/978-94-011-5232-7_28

[B20] Hegazi N. A. Sarhan M. S. Fayez M. Patz S. Murphy B. R. Ruppel S. (2017). Plant-fed versus chemicals-fed rhizobacteria of Lucerne: plant-only teabags culture media not only increase culturability of rhizobacteria but also recover a previously uncultured *Lysobacter* spp., *Novospphingobium* spp. and *Pedobacter* spp. *PLoS One* 12:e00180424 10.1371/journal.pone.0180424 PMC5501534 28686606

[B21] Huschek D. Witzel K. (2019). Rapid dereplication of microbial isolates using matrix assisted laser desorption ionization time-of-flight mass spectrometry: a mini-review. *J. Adv. Res.* 19 99–104. 10.1016/j.jare.2019.03.007 31341675 PMC6629721

[B22] Huson D. A. Scornavacca C. E. (2012). Dendroscope 3: an interactive tool for rooted phylogenetic trees and networks. *Syst. Biol.* 61 1061–1067. 10.1093/sysbio/sys062 22780991

[B23] Ingham C. J. Spprenkels A. Bomer J. Molenaar D. van den Berg A. van Hylckama Vlieg J. E. T. (2007). The micro-Petri dish, a million-well growth chip for the culture and high-throughput screening of microorganisms. *Proc. Natl. Acad. Sci. U.S.A.* 104 18217–18222. 10.1073/pnas.0701693104 17989237 PMC2084323

[B24] Ingham C. J. Van Den Ende M. Pijnenburg D. Wever P. C. Schneeberger P. M. (2005). Growth and multiplexed analysis of microorganisms on a subdivided, highly porous, inorganic chip manufactured from anopore. *Appl. Environ. Microbiol.* 71 8978–8981. 10.1128/AEM.71.12.8978-8981.2005 16332904 PMC1317464

[B25] Jackson C. R. Randolph K. C. Osborn S. L. Tyler H. L. (2013). Culture dependent and independent analysis of bacterial communities associated with commercial salad leaf vegetables. *BMC Microbiol.* 13:274 10.1186/1471-2180-13-274 PMC4219373 24289725

[B26] Jensen V. (1962). Studies on the microflora of Danish beech forest soils. I. The dilution plate count technique for the enumeration of bacteria and fungi in soil. *Zentbl. Bakteriol. Parasitenkd.* 2 13–32.

[B27] Jiang C. Y. Dong L. Zhao J. K. Hu X. Shen C. Qiao Y. (2016). High-throughput single-cell cultivation on microfluidic streak plates. *Appl. Environ. Microbiol.* 82 2210–2218. 10.1128/AEM.03588-15 26850294 PMC4807504

[B28] Jung D. Aoi Y. Epstein S. S. (2016). In situ cultivation allows for recovery of bacterial types competitive in their natural environment. *Microb. Environ.* 31 456–459. 10.1264/jsme2.ME16079 PMC5158119 27682804

[B29] Kaeberlein T. Lewis K. Epstein S. S. (2002). Isolating “Uncultivable” microorganisms in pure culture in a simulated natural environment. *Science* 296 1127–1129. 10.1126/science.1070633 12004133

[B30] Koberl M. Muller H. Ramadan E. M. Berg G. (2011). Desert farming benefits from microbial potential in arid soils and promotes diversity and plant health. *PLoS One* 6:e24452 10.1371/journal.pone.0024452 PMC3166316 21912695

[B31] Kumar S. Stecher G. Tamura K. (2016). MEGA7: Molecular Evolutionary Genetics Analysis Version 7.0 for bigger datasets brief communication. *Mol. Biol. Evol.* 33 1870–1874. 10.1093/molbev/msw054 27004904 PMC8210823

[B32] Leach J. E. Triplett L. R. Argueso C. T. Trivedi P. (2017). Review-communication in the phytobiome. *Cell* 169 587–596. 10.1016/j.cell.2017.04.025 28475891

[B33] Leff J. W. Lynch R. C. Kane N. C. Fierer N. (2016). Plant domestication and the assembly of bacterial and fungal communities associated with strains of the common sunflower, *Helianthus annuus*. *New Phytol.* 214 412–423. 10.1111/nph.14323 27879004

[B34] Letunic I. Bork P. (2007). Interactive tree of life (iTOL): an online tool for phylogenetic tree display and annotation. *Bioinformatics* 23 127–128. 10.1093/bioinformatics/btl529 17050570

[B35] Lindow S. E. Brandl M. T. (2003). Microbiology of the phyllosphere. *Appl. Environ. Microbiol.* 69 1875–1883. 10.1128/AEM.69.4.1875 12676659 PMC154815

[B36] Liu X. Li X. Li Y. Li R. Xie Z. (2017). Plant growth promotion properties of bacterial strains isolated from the rhizosphere of the Jerusalem artichoke (*Helianthus tuberosus* L.) adapted to salt-alkaline soils and their effect on wheat growth. *Can. J Microbiol.* 63 228–237. 10.1139/cjm-2016-0511 28177802

[B37] Manome A. Zhang H. Tani Y. Katsuragi T. (2001). Application of gel microdroplet and fow cytometry techniques to selective enrichment of non-growing bacterial cells. *FEMS Microbiol. Lett.* 197 29–33. 10.1111/j.1574-6968.2001.tb10578.x 11287142

[B38] Marasco R. Rolli E. Ettoumi B. Vigani G. Mapelli F. Borin S. (2012). A drought resistance-promoting microbiome is selected by root system under desert farming. *PLoS One* 7:e48479 10.1371/journal.pone.0048479 PMC3485337 23119032

[B39] Morris R. M. Rappé M. S. Connon S. A. Vergin K. L. Siebold W. A. Carlson C. A. (2002). SAR11 clade dominates ocean surface bacterioplankton communities. *Nature* 420 806–810. 10.1038/nature01240 12490947

[B40] Mühling M. Woolven-Allen J. Murrell J. C. Joint I. (2008). Improved group-specific PCR primers for denaturing gradient gel electrophoresis analysis of the genetic diversity of complex microbial communities. *ISME J.* 2 379–392. 10.1038/ismej.2007.97 18340335

[B41] Nichols D. Cahoon N. Trakhtenberg E. M. Pham L. Mehta A. Belanger A. (2010). Use of ichip for high-throughput *in situ* cultivation of “uncultivable” microbial sppecies. *Appl. Environ. Microbiol.* 76 2445–2450. 10.1128/AEM.01754-09 20173072 PMC2849220

[B42] Nichols D. Lewis K. Orjala J. Mo S. Ortenberg R. O’Connor P. (2008). Short peptide induces an “uncultivable” microorganism to grow *in vitro*. *Appl. Environ. Microbiol.* 74 4889–4897. 10.1128/AEM.00393-08 18515474 PMC2519364

[B43] Oberholster T. Vikram S. Cowan D. Valverde A. (2018). Science of the total environment key microbial taxa in the rhizosphere of sorghum and sunflower grown in crop rotation. *Sci. Total Environ.* 624 530–539. 10.1016/j.scitotenv.2017.12.170 29268225

[B44] Raval A. A. Desai P. B. (2012). Rhizobacteria from rhizosphere of sunflower (*Helianthus annuu*s L.) and their effect on plant growth. *Res. J. Recent Sci.* 1 58–61.

[B45] Reasoner D. J. Geldreich E. E. (1985). A new medium for the enumeration and subculture of bacteria from potable water. *Appl. Environ. Microbiol.* 49 1–7. 10.1016/s0016-7037(99)00322-1 3883894 PMC238333

[B46] Redford A. J. Fierer N. (2009). Bacterial succession on the leaf surface: a novel system for studying successional dynamics. *Microb. Ecol.* 58 189–198. 10.1007/s00248-009-9495-y 19221834

[B47] Sánchez-Juanes F. Ferreira L. Alonso de la Vega P. Valverde A. Barrios M. L. Rivas R. (2013). MALDI-TOF mass sppectrometry as a tool for differentiation of *Bradyrhizobium* sppecies: application to the identification of *Lupinus* nodulating strains. *Syst. Appl. Microbiol.* 36 565–571. 10.1016/j.syapm.2013.09.003 24168963

[B48] Sarhan M. S. Hamza M. A. Youssef H. H. Patz S. Becker M. Elsawey H. (2019). Culturomics of the plant prokaryotic microbiome and the dawn of plant-based culture media – a review. *J. Adv. Res.* 19 15–27. 10.1016/j.jare.2019.04.002 31341666 PMC6630032

[B49] Sarhan M. S. Mourad E. F. Hamza M. A. Youssef H. H. Scherwinski A. C. El-Tahan M. (2016). Plant powder teabags: a novel and practical approach to resolve culturability and diversity of rhizobacteria. *Physiol. Plant.* 157 403–413. 10.1111/ppl.12469 27178359

[B50] Sarhan M. S. Mourad E. F. Nemr R. A. Abdelfadeel M. R. Daanaa H. A. Youssef H. H. (2020). An inoculum-dependent culturing strategy (IDC) for the cultivation of environmental microbiomes and the isolation of novel endophytic Actinobacteria. *J. Antibiot.* 73 66–71. 10.1038/s41429-019-0226-4 31467444 PMC8075983

[B51] Sarhan M. S. Patz S. Hamza M. A. Youssef H. H. Mourad E. F. Fayez M. (2018). G3 phylochip analysis confirms the promise of plant-based culture media for unlocking the composition and diversity of the maize root microbiome and for recovering unculturable candidate divisions/phyla. *Microb Environ.* 33 317–325. 10.1264/jsme2.ME18023 PMC6167109 30210099

[B52] Sauget M. Valot B. Bertrand X. Hocquet D. (2017). Can MALDI-TOF Mass Spectrometry reasonably type bacteria? *Trends Microbiol.* 25 447–455. 10.1016/j.tim.2016.12.006 28094091

[B53] Soussi A. Ferjani R. Marasco R. Guesmi A. Cherif H. (2015). Plant-associated microbiomes in arid lands: diversity, ecology and biotechnological potential. *Plant Soil* 405 357–370. 10.1007/s11104-015-2650-y

[B54] Starostin K. V. Demidov E. A. Bryanskaya A. V. Efimov V. M. Rozanov A. S. Peltek S. E. (2015). Identification of *Bacillus* strains by MALDI TOF MS using geometric approach. *Sci. Rep.* 5 1–9. 10.1038/srep16989 PMC4655313 26592761

[B55] Thompson J. D. Higgins D. G. Gibson T. J. (1994). CLUSTAL W: improving the sensitivity of progressive multiple sequence alignment through sequence weighting, position-specific gap penalties and weight matrix choice. *Nucleic Acids Res.* 22 4673–4680. 10.1093/nar/22.22.4673 7984417 PMC308517

[B56] Verslues P. E. Agarwal M. Katiyar-Agarwal S. Zhu J. Zhu J. K. (2006). Methods and concepts in quantifying resistance to drought, salt and freezing, abiotic stresses that affect plant water status. *Plant J.* 45 523–539. 10.1111/j.1365-313x.2005.02593.x 16441347

[B57] Wang Q. Garrity G. M. Tiedje J. M. Cole J. R. Al W. E. T. (2007). Naïve bayesian classifier for rapid assignment of rRNA sequences into the new bacterial taxonomy. *Appl. Environ. Microbiol.* 73 5261–5267. 10.1128/AEM.00062-07 17586664 PMC1950982

[B58] Watve M. Shejval V. Sonawane C. Rahalkar M. Matapurkar A. Shouche Y. (2000). The “K” selected oligophilic bacteria: a key to uncultured diversity? *Curr. Sci.* 78 1535–1542. 10.1177/0022487110369555

[B59] Whitlow T. H. Bassuk N. L. Ranney T. G. Reichert D. L. (1992). An improved method for using electrolyte leakage to assess membrane competence in plant tissues. *Plant Physiol.* 98 198–205. 10.1104/pp.98.1.198 16668614 PMC1080169

[B60] Yoon S. Ha S. Kwon S. Lim J. Kim Y. Seo H. (2017). Introducing EzBioCloud: a taxonomically united database of 16S rRNA gene sequences and whole-genome assemblies. *Int. J. Syst. Evol. Microbiol.* 67 1613–1617. 10.1099/ijsem.0.001755 28005526 PMC5563544

[B61] Youssef H. H. Fayez M. Monib M. Hegazi N. (2004). *Gluconacetobacter diazotrophicus*: a natural endophytic diazotroph of Nile Delta sugarcane capable of establishing an endophytic association with wheat. *Biol. Fertil. Soils* 39 391–397. 10.1007/s00374-004-0728-4

[B62] Zengler K. Toledo G. Rappe M. Elkins J. Mathur E. J. Short J. M. (2002). Cultivating the uncultured. *Proc. Natl. Acad. Sci. U.S.A.* 99 15681–15686. 10.1073/pnas.252630999 12438682 PMC137776

