# Supplementary material for: “In situ similis” Culturing of Plant Microbiota: A Novel Simulated Environmental Method Based on Plant Leaf Blades as Nutritional Pads
Source: Front Microbiol. 2020 Apr 7;11:454. doi: 10.3389/fmicb.2020.00454 (PMC7154060; doi:10.3389/fmicb.2020.00454)
Supplement: Supplementary file 1 [file Data_Sheet_1.docx]

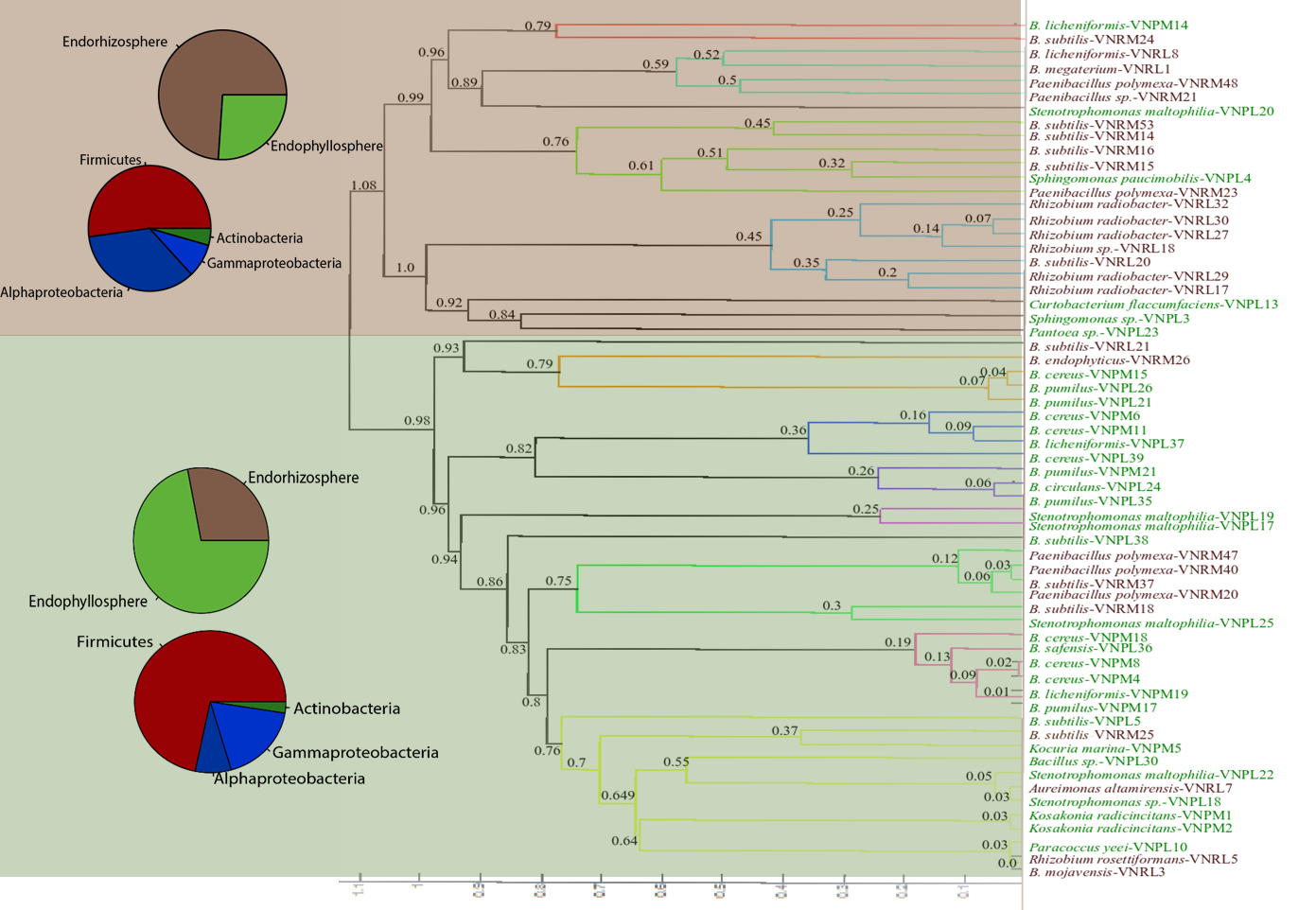
**Figure S1 Cluster analysis of MALDI-TOF spectra of all tested isolates recovered from sunflower endo-rhizosphere (written in purple), and endo-phyllosphere (written in green), isolated either on leaf-surface-inoculated culture medium or leaf-membrane filter culture medium. Distinguished are two main clusters, the first in brownish background represents isolates mainly originated from roots, while the second in greenish background shows isolates mainly originated from the phyllosphere; Pie charts represent percentages of distribution based on either plant sphere or phylum level. Species names are putative/potential being based on partial 16S rRNA gene sequencing, and we should rely on names of genera with caution.**

**Table S1 Number of randomly picked colonies representing CFUs of various plant compartments (endo-rhizosphere and endo-phyllosphere) developed on all tested culture media, and further subcultured and 16S rRNA gene sequenced.**

| **Plant compartments/ Culture Media** | **Total picked colonies** | **Successfully sub-cultured colonies** | **Good quality sequence** |
| --- | --- | --- | --- |
| Endo-rhizosphere/Leaf surface (LS) | 52 | 32 | 20 |
| Endo- rhizosphere/Membrane filter (MF) | 66 | 58 | 21 |
| Endo- rhizosphere/R2A | 35 | 19 | 19 |
| Endo-phyllosphere/Leaf surface (LS) | 83 | 41 | 23 |
| Endo-phyllosphere/Membrane filter (MF) | 33 | 21 | 18 |
| Endo-phyllosphere/R2A | 64 | 52 | 21 |
| **Total** | **333** | **223** | **122** |

**Table S3 Phylogenetic affiliation of total isolates recovered on leaf-based culture media (leaf surface inoculated, LS; leaf-membrane filter, MF methods) and R2A-standard culture medium on the basis of 16S rRNA gene sequencing.**

***Species names are putative/potential being based on partial 16S rRNA gene sequencing, and we should rely on names of genera with caution.**

| **Culture Media** | **Taxonomic group** | **Closest species** | **Similarity (%)** | **No. of isolates** |
| --- | --- | --- | --- | --- |
| R2A-standard culture medium | Firmicutes | *Bacillus subtilis* | 100 | 16 |
|  |  | *Bacillus valenzensis* | 99-100 | 16 |
|  |  | *Bacillus vallismortis* | 100 | 7 |
|  |  | *Bacillus tequilensis* | 100 | 1 |
| Leaf-surface-inoculated method, LS | Firmicutes | *Bacillus subtilis* | 99-100 | 5 |
|  |  | *Bacillus pumilus* | 99-100 | 4 |
|  |  | *Bacillus cereus* | 99 | 1 |
|  |  | *Bacillus aerophilus* | 100 | 1 |
|  |  | *Bacillus circulans* | 99 | 1 |
|  |  | *Bacillus licheniformis* | 97-99 | 2 |
|  |  | *Bacillus megaterium* | 99 | 1 |
|  |  | *Bacillus mojavensis* | 99 | 1 |
|  |  | *Bacillus safensis* | 100 | 2 |
|  | Alphaproteobacteria | *Rhizobium rosettiformans* | 99 | 1 |
|  |  | *Rhizobium radiobacter* | 99 | 11 |
|  |  | *Aureimonas altamirensis* | 99 | 1 |
|  |  | *Sphingomonas paucimobilis* | 99 | 1 |
|  |  | *sphingomonas yanoikuyae* | 99 | 1 |
|  |  | *Paracoccus yeei* | 100 | 1 |
|  | Gammaproteobacteria | *Stenotophomonas maltophilia* | 99 | 5 |
|  |  | *stenotophomonas pavanii* | 99 | 1 |
|  |  | *Pantoea agglomerans* | 98 | 1 |
|  | Actinobacteria | *Curtobacterium flaccumfaciens* | 100 | 1 |
|  |  | *Microbacterium paraoxydans* | 99 | 1 |
| Leaf-membrane filter method, MF | Firmicutes | *Bacillus subtilis* | 99-100 | 9 |
|  |  | *Bacillus endophyticus* | 99 | 2 |
|  |  | *Bacillus licheniformis* | 99 | 2 |
|  |  | *Bacillus cereus* | 99 | 9 |
|  |  | *Bacillus pumilus* | 99-100 | 2 |
|  |  | *Paenibacillus timonensis* | 99 | 1 |
|  |  | *Paenibacillus polymyxa* | 99 | 9 |
|  | Gammaproteobacteria | *Kosakonia radicincitans* | 99 | 2 |
|  |  | *Kosakonia oryzae* | 99 | 1 |
|  |  | *Erwinia sp.* | 98 | 1 |
|  | Actinobacteria | *Kocuria marina* | 99 | 1 |
